# Effects of Transcranial Direct Current Stimulation on Core Symptoms and Sleep Disturbances in Children With Autism Spectrum Disorder: A Meta‐Analysis of Randomized Controlled Trials

**DOI:** 10.1002/brb3.71918

**Published:** 2026-11-09

**Authors:** Mingchen Wang, Meimei Yang

**Affiliations:** ^1^ Tianjin University of Traditional Chinese Medicine Tianjin China; ^2^ College of Traditional Chinese Medicine Chongqing Three Gorges Medical College Chongqing China

**Keywords:** autism spectrum disorder, children, meta‐analysis, sleep disorders, transcranial direct current stimulation

## Abstract

**Objective:**

This meta‐analysis was designed to determine whether transcranial direct current stimulation (tDCS) confers measurable benefits for selected behavioral features of autism spectrum disorder (ASD) and for sleep problems in pediatric ASD populations.

**Methods:**

A comprehensive literature retrieval was conducted across eight electronic databases—PubMed, Cochrane Library, Web of Science, Embase, CNKI, CBM, VIP, and Wanfang—covering the period from each database's inception through June 1, 2026. Eligible studies were randomized controlled trials (RCTs) that evaluated the therapeutic efficacy of transcranial direct current stimulation (tDCS) in patients with autism spectrum disorder (ASD). The methodological quality of the included trials was appraised using the Cochrane Risk of Bias assessment instrument. Quantitative synthesis was performed with RevMan 5.4 and Stata 17.0, following the PRISMA statement for systematic reviews and meta‐analyses.

**Results:**

Ultimately, seven studies qualified for inclusion in this systematic review. Pooled analyses showed that tDCS was associated with lower ATEC scores (mean difference [MD] = −8.81, 95% confidence interval [CI]: −12.06 to −5.56, *p* < 0.00001), ABC scores (MD = −12.08, 95% CI: −21.75 to −2.41, *p* = 0.01), and CSHQ scores (MD = −4.95, 95% CI: −8.66 to −1.24, *p* = 0.009). However, no statistically significant difference was observed for CARS scores (MD = −1.12, 95% CI: −4.15 to 1.91, *p* = 0.47).

**Conclusion:**

tDCS, most commonly delivered at 1 mA over the DLPFC, was associated with improvements in some ASD‐related behavioral outcomes; preliminary evidence also suggested a potential benefit for sleep disturbances, although this finding was based on only two low‐certainty studies and warrants cautious interpretation.

## 1 Introduction

Autism spectrum disorder (ASD) represents a neurodevelopmental disorder characterized by core impairments encompassing persistent deficits in social communication and reciprocal social interaction, alongside constrained or stereotyped patterns of behavior and interests. Nevertheless, these manifestations often remain undetected until environmental or social expectations surpass the individual's adaptive capacity, and ASD‐related symptoms generally lead to substantial functional impairment spanning several domains. According to surveillance estimates from the Centers for Disease Control and Prevention (CDC) via the Autism and Developmental Disabilities Monitoring (ADDM) Network, roughly 1 in 31 children at 8 years of age (3.2%) are diagnosed with ASD. At present, the prevalence of ASD among boys (49.2 per 1,000) is 3.4 times that observed among girls (14.3 per 1,000) (Shaw et al. 2025). Such an upward trend in prevalence could signify not merely an actual growth in ASD incidence but may equally be attributable to refinements in diagnostic criteria or advances in assessment instruments (Sharma et al. 2018). The etiology of ASD is multifactorial, with genetic and environmental determinants each contributing critically to the pathogenesis of the disorder (Sharma et al. 2018). Pathological studies on ASD have found differences in brain development, structure, and connectivity in individuals with the condition. The mechanism of action is complex, and effective clinical treatments are still lacking.

Recent studies have shown promising results in treating ASD using non‐invasive brain stimulation techniques (Khaleghi et al. 2020), with one of the main techniques being tDCS. This method applies a current through electrodes placed on the scalp (Fernández‐Corazza et al. 2020). During tDCS, a low‐intensity electrical current is applied via electrodes positioned on the scalp, which modifies neuronal membrane potentials and consequently changes spontaneous firing rates (Sandrini et al. 2020). In a two‐electrode configuration, one electrode functions as the anode while the other acts as the cathode. Anodal polarization enhances neuronal excitability through depolarization, whereas cathodal polarization reduces excitability via hyperpolarization. This approach is capable of modulating the excitability of distinct cortical regions, and its influence on neuronal excitability may persist for minutes to hours following extended stimulation (Dalong et al. 2020; Li et al. 2019). Typically, a single tDCS session lasts no more than 20 min, employing a current strength of 1–2 mA. Under such parameters, tDCS has demonstrated a favorable safety profile and good tolerability (Zewdie et al. 2020). This form of brain stimulation has been tested as a therapeutic option in various psychiatric disorders, and tDCS appears to have positive effects on depression and certain symptoms of schizophrenia (Borrione et al. 2022; Narita et al. 2020), particularly in areas such as inhibitory control and working memory (Salehinejad et al. 2019). Increasing evidence suggests that social, cognitive, and sensory dysfunctions in ASD are associated with abnormalities in distributed networks rather than isolated brain regions. Therefore, tDCS may produce favorable effects due to its ability to modulate functional connectivity in the brain. Nevertheless, although tDCS has attracted increasing attention in autism research, its effects on sleep problems remain poorly characterized. Sleep disturbances are very common in children with autism and can have a big impact on their quality of life. A lot of people have sleep problems, and this makes them act in a worse way and makes them find it harder to think. Some studies have looked at how tDCS can help with the main symptoms of ASD, but there is not much evidence that it can improve sleep problems in children with ASD.

Against this backdrop, the present meta‐analysis seeks to fill this gap by systematically assessing the efficacy of tDCS for alleviating ASD symptoms, with particular emphasis on its influence on sleep disturbances within this population. This will help clinicians and researchers understand better how tDCS could be used to treat not only core behavioral symptoms but also sleep problems, which are a clinical challenge that hasn't been fully addressed yet. Although some reviews have explored this area, there is a lack of objective, evidence‐based conclusions (Osório and Brunoni 2019). This means that a meta‐analysis is needed to evaluate the efficacy of tDCS in treating ASD.

## 2 Materials and Methods

This systematic review and meta‐analysis was prospectively registered with the PROSPERO database (registration no. CRD42024588423). Its design and reporting conformed rigorously to the Preferred Reporting Items for Systematic Reviews and Meta‐Analyses (PRISMA) guidelines, thereby guaranteeing transparency and comprehensiveness.

### 2.1 Inclusion Criteria for Studies

#### 2.1.1 Study Design

Randomized controlled trials (RCTs) investigating tDCS in pediatric ASD populations and published in either Chinese or English were eligible.

#### 2.1.2 Participants

Individuals with a definitive ASD diagnosis established according to the Diagnostic and Statistical Manual of Mental Disorders, Fifth Edition (DSM‐5) (American Psychiatric Association 2013) or the Chinese Classification of Mental Disorders, Third Edition (CCMD‐3).

#### 2.1.3 Intervention

While the control arm received sham stimulation or standard rehabilitation, the experimental arm received tDCS in addition to routine care.

#### 2.1.4 Outcome Measures

Core ASD symptoms—including social interaction, language ability, perception/cognition, and health/behavior—were evaluated using the Autism Treatment Evaluation Checklist (ATEC), where higher scores denote greater symptom severity and lower scores indicate milder manifestations. The Autism Behavior Checklist (ABC) serves as a preliminary instrument for assessing social interaction, communication, stereotyped behaviors, and interests in children with ASD. The Childhood Autism Rating Scale (CARS) is employed to facilitate the diagnosis and evaluation of ASD in children and to track therapeutic response. The Children's Sleep Habits Questionnaire (CSHQ) was designed primarily to determine the presence, characteristics, and severity of sleep problems in children. For inclusion, studies needed to report pre‐ and post‐intervention means and standard deviations (SD) for these scales in both the tDCS and sham groups.

### 2.2 Exclusion Criteria for Studies

Empirical studies such as reviews, conference proceedings, and anecdotal reports. Cohort, case‐control, and cross‐sectional study designs were excluded. Likewise, articles published in languages other than English or Chinese were not considered.

### 2.3 Literature Search

A total of eight electronic databases—PubMed, Cochrane Library, Web of Science, Embase, CNKI, CBM, VIP, and Wanfang—were searched, encompassing all records available from their inception through June 1, 2026. To maximize the identification of pertinent studies, a customized search strategy integrating medical subject headings (MeSH) with free‐text terms was employed for each database.

In PubMed, the search combined MeSH terms such as “Autism Spectrum Disorder,” “Transcranial Direct Current Stimulation,” and terms for randomized controlled trials (e.g., “Randomized Controlled Trial,” “Placebo Controlled Trial”), along with corresponding free‐text keywords like “autism,” “tDCS,” and “RCT.”

In Embase, Emtree terms aligned with autism and brain stimulation were used in combination with free‐text terms and filters for clinical trials.

For Chinese databases (CNKI, CBM, VIP, Wanfang), equivalent subject terms and keywords were mapped from Chinese terminology to capture local literature comprehensively.

The exact English search strategy was as follows:

#1: “Autism Spectrum Disorder” OR “Autistic Spectrum Disorder” OR “Autism” OR “Disorder, Autistic Spectrum”
#2: “Transcranial Direct Current Stimulation” OR “Noninvasive Brain Stimulation” OR “Transcranial Electrical Stimulation” OR “Transcranial Direct Current” OR “tDCS” OR “NIBS”
#3: “Randomized Controlled Trial” OR “Controlled Clinical Trial” OR “Randomized” OR “Placebo” OR “RCT” OR “RCTs”
#4: #1 AND #2 AND #3

Study selection and data extraction were performed independently by MingchenWang and Meimei Yang using a standardized form. Any disagreements concerning study eligibility or data interpretation were initially addressed through discussion between the two reviewers. When a consensus remained unattainable, Mingchen Wang was consulted as the third reviewer to render the final decision. This process ensured that study selection and data extraction were rigorous and minimized bias.

### 2.4 Outcome Measures

Two researchers independently screened the studies against the predefined inclusion and exclusion criteria. The screening process began with an assessment of titles and abstracts, followed by a full‐text review. Any disagreements that arose were settled through discussion. The following variables were extracted: (1) first author and publication year; (2) demographic information (number of patients, country, and age); (3) means and standard deviations (SD) for the following scale scores: (I) ABC, ATEC, CARS, and CSHQ; and (II) tDCS parameters (intensity, location, and treatment duration). Summary data from these RCTs were independently extracted by two researchers.

### 2.5 Quality Assessment of Literature

The Cochrane Handbook version 5.1.0 (Higgins et al. 2019) served as the basis for appraising methodological quality, as it provides overarching recommendations regarding the reporting of RCTs. This earlier version of the Cochrane risk‐of‐bias tool was selected, rather than the current RoB 2 tool, because RoB 2 requires domain‐level signaling questions that were not consistently reported across the included trials; we acknowledge this as a methodological limitation and address its implications for the certainty of evidence in the discussion. For any quantitative synthesis that incorporated two or more studies, publication bias was evaluated based on the *p*‐value derived from Egger's test. In accordance with the quality assessment criteria specified in the handbook, risk was classified into three categories: unclear, low, and high risk. Four authors were responsible for assessing the quality of the literature, and cross‐checking was performed to ensure accuracy, resulting in the final risk classification.

### 2.6 Statistical Analysis

Meta‐analytic calculations were performed using RevMan 5.4 and Stata 17.0. Continuous data were summarized as the mean difference (MD) or standardized mean difference (SMD), with 95% confidence intervals computed for each estimate. Heterogeneity across studies was quantified using the I^2^ value together with the *p*‐value. The selection between fixed‐ and random‐effects models depended on the heterogeneity parameters: when I^2^ < 50% and *p* > 0.1, a fixed‐effects model was selected; when I^2^ ≥ 50% and *P* ≤ 0.1, a random‐effects model was chosen. Substantial heterogeneity was defined by I^2^ ≥ 50% combined with *P* ≤ 0.1, and under such circumstances a random‐effects model was utilized. Egger's test was conducted to detect publication bias when the analysis comprised two or more studies. Statistical significance was determined at *p* < 0.05, with *P* ≥ 0.05 indicating a non‐significant result. A Z‐test was employed to determine the overall effect of tDCS, and bilateral *P*‐values were derived. The significance thresholds were established a priori: *p* < 0.05 for the overall effect and *p* < 0.10 for tests of heterogeneity (χ^2^ and subgroup interactions). Publication bias was assessed with the Egger linear regression test in Stata 17.0 (StataCorp, College Station, Texas, USA) whenever four or more studies contributed to the analysis of a particular outcome. To investigate potential sources of heterogeneity, subgroup analyses were carried out, and chi‐squared (χ^2^) tests were used to evaluate subgroup interactions. The stability of the pooled estimates was tested by sensitivity analyses that involved the sequential removal of each individual study.

### 2.7 GRADE Quality of Evidence Assessment

For studies that reported two or more outcome measures, the quality of evidence was appraised. Evidence quality was evaluated using the GRADE approach and classified as high, moderate, low, or very low on the basis of limitations, heterogeneity, bias, indirectness, and publication bias.

## 3 Results

### 3.1 Literature Screening Results

A total of 568 records were retrieved through the search. Following the removal of 307 duplicates with NoteExpress, 261 articles were left. Of these, 232 were eliminated during title and abstract screening. Full‐text assessment according to the screening criteria resulted in the exclusion of an additional 22 articles, leaving 7 studies eligible for the final analysis. The screening workflow and detailed results are illustrated in Figure 1.

**FIGURE 1:**
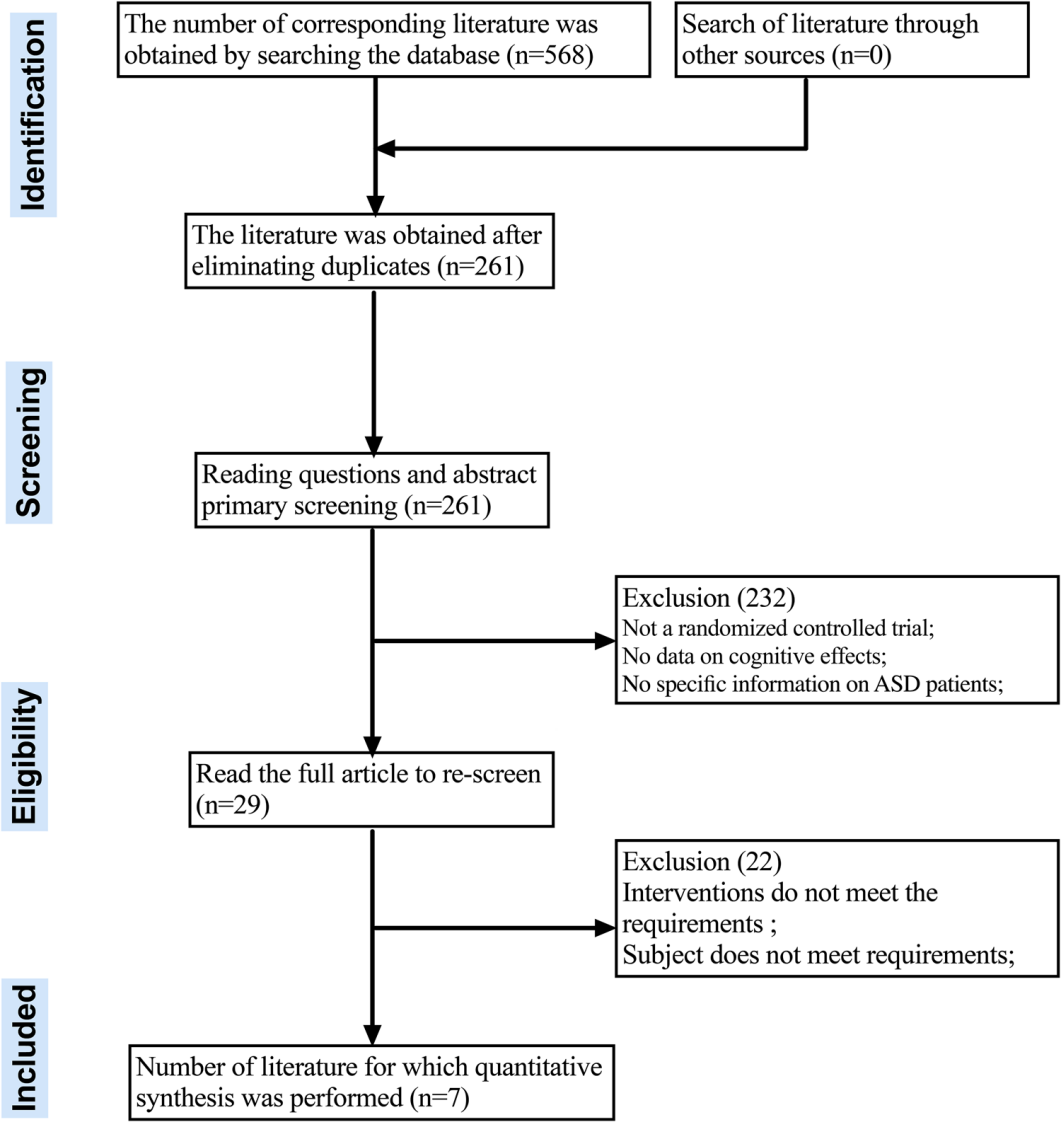
Literature screening flowchart.

### 3.2 Quality Assessment of Included Studies

Of the seven eligible studies, all (Amatachaya et al. 2014; Amatachaya et al. 2015; Hadoush et al. 2020; Tian 2020; Qiu et al. 2021; Sun et al. 2021; Jin 2023) reported random allocation, and three (Tian 2020; Sun et al. 2021; Jin 2023) explicitly stated the use of random number tables. Allocation concealment was not described in any study. Blinding of researchers and participants was reported in five studies (Amatachaya et al. 2014; Amatachaya et al. 2015; Hadoush et al. 2020; Tian 2020; Qiu et al. 2021), whereas the remaining two (Sun et al. 2021; Jin 2023) made no mention of blinding. Complete data with detailed results were provided by all seven studies, and no other sources of bias were identified. Further specifics are presented in Figure 2.

**FIGURE 2:**
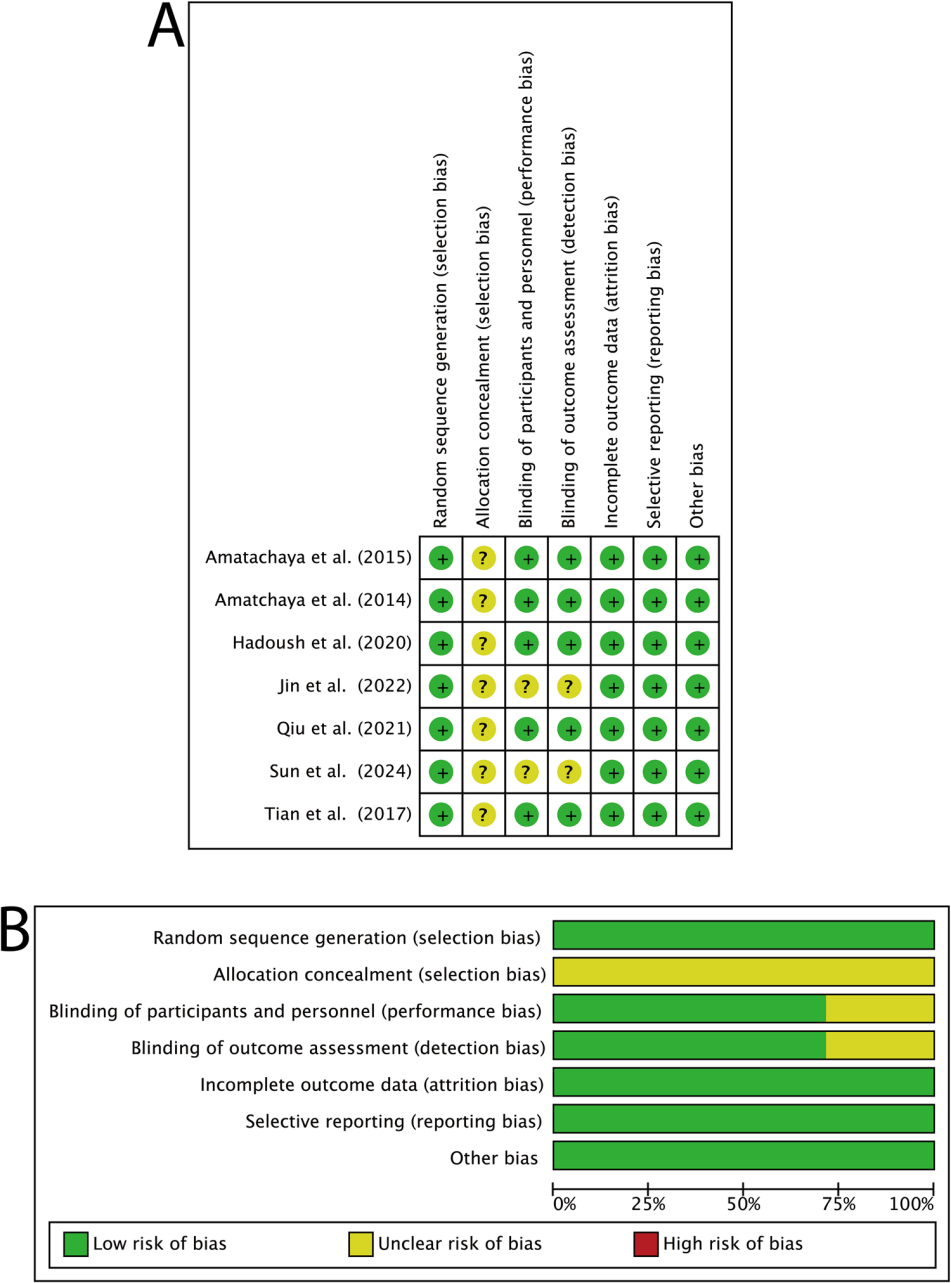
Quality assessment of the included studies. (A) Risk of bias graph; (B) Risk of bias summary.

### 3.3 Characteristics of Included Studies

Seven studies (Amatachaya et al. 2014; Amatachaya et al. 2015; Hadoush et al. 2020; Tian 2020; Qiu et al. 2021; Sun et al. 2021; Jin 2023) were ultimately included, comprising three Chinese studies (Tian 2020; Sun et al. 2021; Jin 2023) and four foreign studies (Amatachaya et al. 2014; Amatachaya et al. 2015; Hadoush et al. 2020; Qiu et al. 2021), with a total of 235 children diagnosed with ASD. In the majority of studies, the mean age of participants exceeded 5 years. Autism treatment evaluations were conducted on 169 patients, autism behavior checklists on 150 patients, childhood autism rating scales on 80 patients, and children's sleep habits questionnaires on 86 patients. Four studies (Amatachaya et al. 2014; Amatachaya et al. 2015; Hadoush et al. 2020; Jin 2023) used tDCS to assess ATEC scores in children with ASD, four studies (Tian 2020; Qiu et al. 2021; Sun et al. 2021; Jin 2023) assessed ABC scores, two studies (Amatachaya et al. 2014; Qiu et al. 2021) evaluated CARS scores, and two studies (Qiu et al. 2021; Jin 2023) assessed CSHQ scores. In the majority of studies, the anode was positioned over the DLPFC, while the cathode was placed on the contralateral right shoulder. Most patients in these trials were receiving stable rehabilitation both before and throughout the tDCS intervention, thereby maintaining their rehabilitation status. Table 1 provides detailed information regarding the patients and the tDCS parameters adopted in these studies.

**TABLE 1:** Characteristics of included studies.

Researcher year	Country	Sample size (C/T)	Age (C/T)	Interventions (C/T)	Stimulation frequency	Stimulation intensity	Stimulation Site (anode/cathode)	Treatment duration	Outcome measures			
Amatachaya (Amatachaya et al. 2014) et al. 2014	Thailand	20	6.42 ± 1.10	①②/tDCS②	1 mA	20 min	DLPFC/Contralateral right shoulder	8 weeks	ATEC	—	CARS	—
Amatachaya (Amatachaya et al. 2015) et al. 2015	Thailand	20	6.40 ± 1.07	①②/tDCS②	1 mA	20 min	DLPFC/Contralateral right shoulder	3 weeks	ATEC	—	—	—
Hadoush et al. 2020 (Hadoush et al. 2020)	Jordan	25/25	8.00 ± 2.80/7.60 ± 2.20	①②/tDCS②	1 mA	20 min	Bilateral tDCS stimulation (left and right prefrontal cortex and motor area)	2 weeks	ATEC	—	—	—
Qiu (Qiu et al. 2021) et al. 2021	Chin	21/22	4.34 ± 1.24/4.35 ± 1.23	①②/tDCS②	1 mA	20 min	DLPFC/Contralateral right shoulder	3 weeks	—	ABC	CARS	CSHQ
Tian Rong (Tian 2020), 2020	Chin	14/7	7.64 ± 2.34/8.12 ± 2.09	①②/tDCS②	1.5 mA	20 min	DLPFC/Contralateral supraorbital region	4 weeks	—	ABC	—	—
Jin Weiwei (Jin 2023), 2023	Chin	23/23	4.18 ± 0.85/4.22 ± 0.79	②/③tDCS	1 mA	20 min	Left DLPFC/Right supraorbital region	12 weeks	ATEC	ABC	—	CSHQ
Sun Changcheng (Sun et al. 2021) et al. 2021	Chin	16/19	7.50 ± 2.22/8.06 ± 1.98	①②/tDCS②	1.5 mA	20 min	DLPFC/ Contralateral supraorbital region	4 weeks	—	ABC	—	—

Abbreviations: C: Control group; DLPFC: Dorsolateral Prefrontal Cortex; T: Treatment group; ①: Sham stimulation; ②: Rehabilitation treatment; ③: Music.

### 3.4 Meta‐Analysis

#### 3.4.1 The Effect of tDCS on ATEC in Children With ASD

Four studies (Amatachaya et al. 2014; Amatachaya et al. 2015; Hadoush et al. 2020; Jin 2023) adopted ATEC as an outcome measure, encompassing 85 patients in the experimental group and 84 in the control group. Given the absence of significant heterogeneity (I^2^ = 12%, *p* = 0.33), a fixed‐effects model was employed. The pooled analysis revealed that tDCS produced a significant improvement in ATEC scores among children with ASD (MD = −8.81, 95% CI −12.06 to −5.56, *p* < 0.001). The results are illustrated in Figure 3A.

**FIGURE 3:**
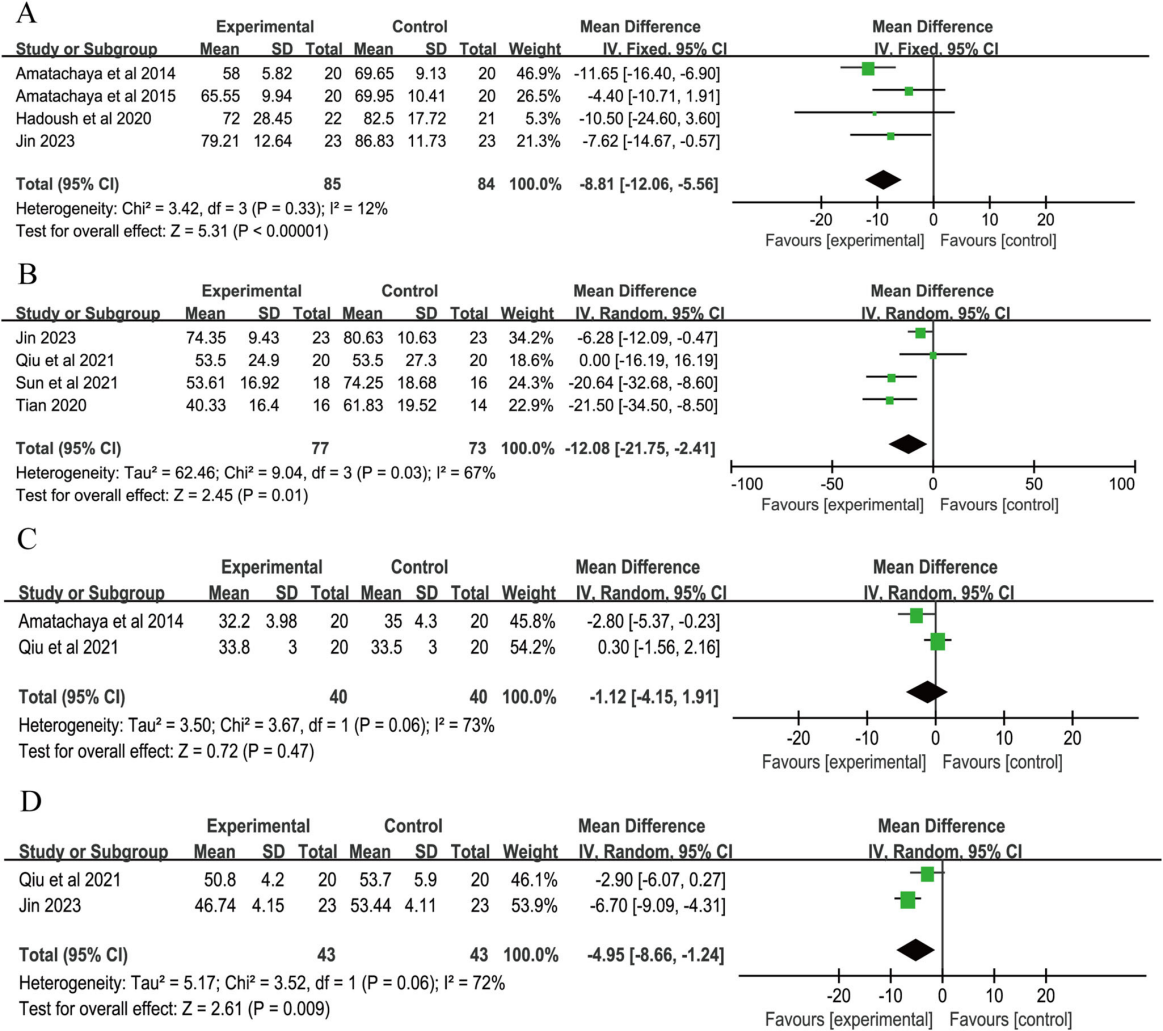
Forest plots of the meta‐analysis results. A: Effect of tDCS on ATEC in children with ASD; B: Effect of tDCS on ABC in children with ASD; C: Effect of tDCS on CARS in children with ASD; D: Effect of tDCS on CSHQ in children with ASD.

#### 3.4.2 The Effect of tDCS on ABC in Children With ASD

ABC was evaluated as an outcome measure in four studies (Tian 2020; Qiu et al. 2021; Sun et al. 2021; Jin 2023), which collectively enrolled 150 patients across both groups. The heterogeneity test revealed significant between‐study differences (MD = −12.08, 95% CI −21.75 to −2.41, *p* = 0.01), warranting the use of a random‐effects model. The pooled findings demonstrated that tDCS exerted a significant beneficial effect on autism‐related behavior in children with ASD. The results are presented in Figure 3B.

#### 3.4.3 The Effect of tDCS on CARS in Children With ASD

CARS was adopted as an outcome measure in two studies (Amatachaya et al. 2014; Qiu et al. 2021), comprising a total of 80 patients (40 in the experimental group and 40 in the control group). Given the significant heterogeneity detected (I^2^ = 73%, *p <* 0.001), a random‐effects model was employed. The pooled analysis revealed no statistically significant difference in CARS scores between the experimental and control groups (MD = −1.12, 95% CI −4.15 to 1.91, *p =* 0.47), as illustrated in Figure 3C. In the subgroup analysis, tDCS at 1.5 mA produced a favorable effect on autistic behavior in children with ASD (SMD = −21.04, 95% CI [−29.87, −12.20], *p <* 0.001), whereas 1 mA tDCS exerted only a modest effect (SMD = −5.56, 95% CI [−11.03, −0.10], *p =* 0.01; Figure 4). These subgroup findings suggest that higher stimulation intensity (1.5 mA) may be associated with greater symptom improvement; however, only two studies used 1.5 mA, limiting the strength of this inference.

**FIGURE 4:**
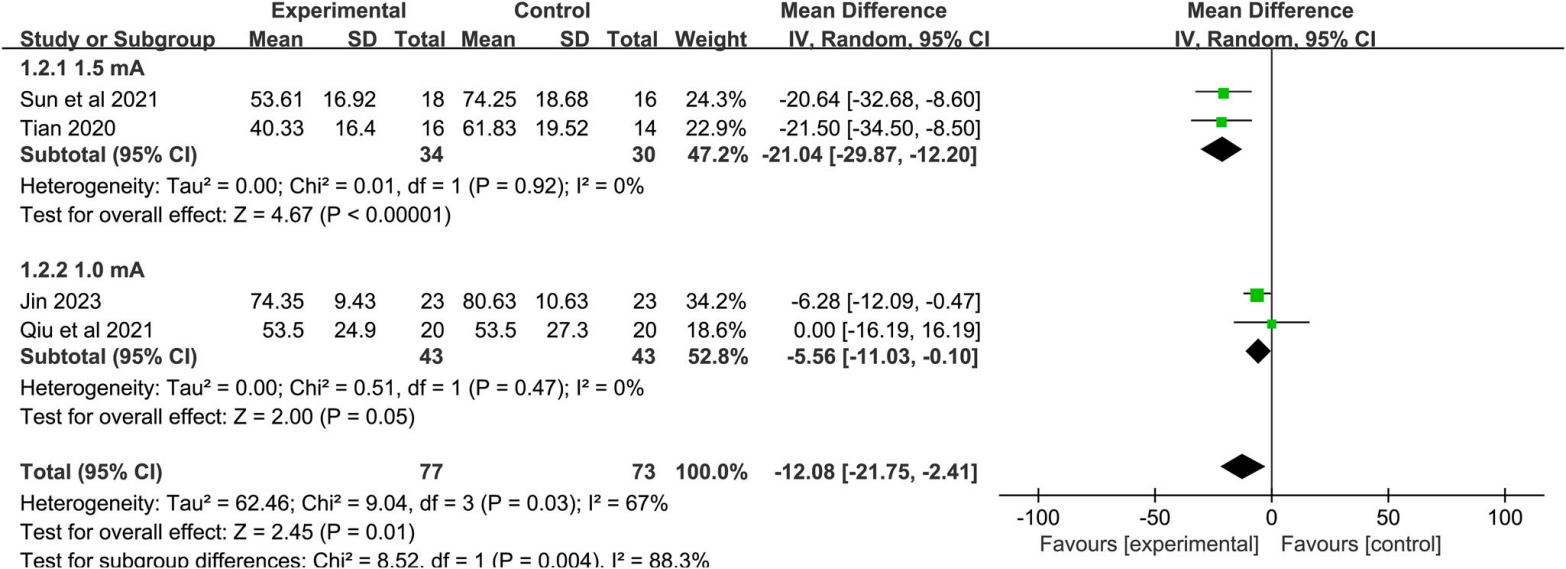
Subgroup analysis of the effect of tDCS on CARS scores in children with ASD.

#### 3.4.4 The Effect of tDCS on CSHQ in Children With ASD

CSHQ data were available from two studies (Qiu et al. 2021; Jin 2023), encompassing 86 patients in total (43 in the experimental group and 43 in the control group). The heterogeneity test revealed a high level of heterogeneity (I^2^ = 72%, *p =* 0.06). Using a random‐effects model, the pooled result showed a statistically significant reduction in CSHQ scores favoring tDCS (MD = −4.95, 95% CI −8.66, ‐1.24, *p =* 0.009); however, given that this estimate is based on only two studies with considerable heterogeneity and was rated as low‐certainty evidence (see Section 3.7), it should be interpreted as preliminary evidence of a potential effect on sleep disturbances rather than a confirmed treatment benefit. See Figure 3D.

### 3.5 Sensitivity Analysis

Sensitivity analysis using the leave‐one‐out approach was conducted. No meaningful shifts in the outcome measures were observed, thereby demonstrating the stability of the pooled results. As a result, meta‐regression and subgroup analysis were not pursued further.

### 3.6 Publication Bias

Egger's test was used for outcomes with at least four studies, and funnel plots were planned for outcomes with at least ten studies. Egger's test results for ATEC (*P* > 0.99) and ABC (*P* > 0.99) were non‐significant; however, with only four studies contributing to each analysis, the statistical power of tests for funnel‐plot asymmetry is very limited, and a non‐significant result cannot be interpreted as evidence for the absence of publication bias. Publication bias for these outcomes should therefore be regarded as unable to be reliably assessed given the small number of studies, rather than as ruled out.

### 3.7 Credibility of Evidence

After conducting GRADE quality assessments for studies reporting ATEC, ABC, CARS, and CSHQ scores, the results showed varying levels of evidence quality across different outcome measures (see Table 2). The ATEC score was based on four studies (total sample size of 169), with no significant issues identified in terms of limitations, indirect evidence, or publication bias, but some uncertainty was found in terms of error, leading to a downgrade in the evidence quality to moderate. The ABC score was also based on four studies (total sample size of 150), with no significant issues related to limitations, indirect evidence, or publication bias, but due to the presence of errors, the evidence quality was downgraded to low. CARS and CSHQ scores were based on two studies each, with relatively small sample sizes (80 and 86 patients, respectively). No significant issues were found regarding limitations and indirect evidence, but due to large errors, the evidence quality for both scores was downgraded to low.

**TABLE 2:** GRADE evidence quality assessment.

Outcome measure	Number of studies	Sample size	Limitations	Heterogeneity	Indirect evidence	Imprecision	Publication bias	Evidence level
ATEC	4	169	0	0	0	Downgraded one level ^b^	0	Moderate
ABC	4	150	0	Downgraded one level ^a^	0	Downgraded one level ^b^	0	Low
CARS	2	80	0	Downgraded one level ^a^	0	Downgraded one level ^b^	0	Low
CSHQ	2	86	0	Downgraded one level ^a^	0	Downgraded one level ^b^	0	Low

*Note*: .

^a^ Significant differences in the overlapping confidence intervals among the studies, with high I^2^ and low *P*‐values in the heterogeneity tests.

^b^ Small sample sizes, leading to wide confidence intervals and significant overlap. c. Evidence of publication bias.

## 4 Discussion

Autism spectrum disorder (ASD) is a multifaceted neurodevelopmental condition whose prevalence continues to rise (Fombonne et al. 2021; Christensen et al. 2016), exerting a substantial impact on the quality of life of both patients and their families (Li et al. 2022). Although pharmacological treatments may offer some relief, they often come with adverse side effects (D'Alò et al. 2021). As the number of children with ASD increases, various therapeutic approaches have been developed and applied clinically. Recent studies suggest that transcranial tDCS shows promising results on ASD‐related behavioral and functional outcomes (Chan et al. 2023), particularly in enhancing social cognition and interaction skills (Wang et al. 2023). This technique not only reduces social pressure on patients but also shortens the time needed for rehabilitation to some extent. As a non‐invasive technique for brain stimulation and neuromodulation, tDCS shows considerable promise in the management of ASD.

The present study consolidates the existing scientific evidence regarding tDCS in individuals with ASD. In contrast to earlier reviews, one recently published trial (Jin 2023) was incorporated, and the pooled meta‐analytic findings indicate that tDCS was linked to improvements in certain ASD‐related behavioral domains—encompassing socialization, sensory and cognitive awareness, health status, and behavioral problems—among children with ASD, as measured by the ATEC and ABC. No significant effect was observed on CARS, which more specifically targets core diagnostic features of ASD. Pooled CSHQ data also suggested a potential beneficial effect on sleep disturbances, but this finding was based on only two studies with considerable heterogeneity and low‐certainty evidence and should be regarded as preliminary rather than an established finding. A polarity comparison study involving ASD and non‐ASD participants revealed that anodal stimulation generally enhances motor cortex excitability in children; however, within the ASD group, anodal stimulation delivered to the same region (M1) led to a reduction in motor evoked potentials (Penton et al. 2019). Direct comparative investigations of anodal versus cathodal stimulation over the same region may help elucidate the efficacy of each polarity in mitigating ASD symptoms. Additionally, this study found that when anodal or cathodal stimulation was applied to the left DLPFC (Rothärmel et al. 2019), cognitive function improved. Studies in both healthy populations and children with ASD have observed improvements in cognitive function (Bueno et al. 2019) and reductions in depression (Moreno et al. 2020) following tDCS. A recent meta‐analysis (Begemann et al. 2020) confirmed the cross‐diagnostic positive effects of tDCS on working memory and attention. Although the overall effect size was small, the differences were statistically significant, suggesting future research on tDCS's effects on children with ASD is warranted.

Although the effect sizes observed in the meta‐analyses were statistically significant, they were generally small, which is an important consideration for clinical practice. Small effect sizes suggest that while tDCS may produce beneficial changes at the group level, the magnitude of improvement for individual patients may be modest. This highlights the need for clinicians to set realistic expectations when considering tDCS as a treatment option for ASD and to balance enthusiasm with caution. In clinical practice, tDCS is unlikely to be used as a stand‐alone therapy but rather as an adjunct to other behavioral and pharmacological interventions. The modest effects highlight the importance of personalized treatment plans that take into account patient‐specific factors such as symptom severity, comorbidities, and responsiveness to stimulation.

The included studies exhibited substantial clinical and methodological heterogeneity that may limit the interpretation and generalizability of the pooled effect estimates. Specifically, participant age ranged from preschool (mean ∼4 years) to school‐age children (mean ∼8 years), stimulation intensity varied between 1 mA and 1.5 mA, electrode montages differed (unilateral vs. bilateral; DLPFC vs. motor cortex), treatment duration ranged from 2 to 12 weeks, and concomitant interventions included routine rehabilitation, music therapy, or no additional treatment. These differences may contribute to the observed heterogeneity in effect sizes and complicate direct comparison across studies.

It should be noted that while the majority of included studies used 1 mA stimulation intensity, our subgroup analysis (Section 3.4.3) indicated that 1.5 mA may produce larger effects on some outcomes. However, with only two studies using 1.5 mA and given the heterogeneity in other protocol parameters (electrode placement, duration, and concomitant interventions), it is premature to recommend a specific optimal intensity. Future dose‐finding studies systematically comparing 1 mA, 1.5 mA, and potentially higher intensities within the same trial design are needed to establish evidence‐based intensity recommendations.

In terms of sleep, the pooled CSHQ analysis showed lower (better) scores in the active tDCS group than in the control group; however, as this analysis is based on only two studies (n = 86) with considerable heterogeneity (I^2^ = 72%) and was rated as low‐certainty evidence, it should be interpreted as preliminary evidence of a potential effect rather than confirmation that tDCS improves sleep quality in children with ASD. According to one study (Amatachaya et al. 2015), EEG activity underwent changes, implying that the elevation in neural activity induced by tDCS could contribute to better sleep. Despite these encouraging findings, studies assessing the impact of tDCS on neuropsychological function and neural activity remain scarce. Future investigations should generate additional evidence in these domains and identify the most useful outcome predictors for tailoring personalized intervention strategies.

Of all tDCS stimulation protocols, the left DLPFC represents the most commonly selected target. This region is regarded as crucial for cognitive control, as it integrates lower‐level motor and sensory processes, thereby enabling individuals to exert voluntary control over their behavior in pursuit of specific goals (MacDonald et al. 2000). Specifically, the DLPFC plays an important role in the maintenance and manipulation of information in working memory, attention shifting, response inhibition (Barbey et al. 2013), social cognition (McDonald et al. 2020), emotional regulation (Kroes et al. 2019), and language processing (Hertrich et al. 2021). Thus, DLPFC involvement may contribute to impairments in some higher‐order functions in individuals with ASD (Herrington et al. 2015). This review noted that investigations targeting the left DLPFC documented improvements in ASD scores (Amatachaya et al. 2014; Amatachaya et al. 2015; Hadoush et al. 2020). Moreover, one study (van Steenburgh et al. 2017) directly compared left versus right anodal stimulation across a battery of neuropsychological tests and observed working memory gains with both protocols, while attention improved only under right anodal stimulation, possibly reflecting the involvement of the right DLPFC in attentional shifting.

This study has several limitations. First, the number of controlled trials in recent years has been relatively limited, and the control conditions vary between studies. Although sham stimulation is the most widely used control condition in trials, indicating a potential for future positive developments, the high heterogeneity between studies, including differences in tDCS protocols and assessment measures, combined with the small number of trials included in this study, make it difficult to compare results effectively. As a result, only 7 studies were suitable for meta‐analysis, far below the recommended standard (Chandler et al. 2019). Second, methodological quality was another important limitation: none of the included trials reported allocation concealment, and two trials did not report blinding of participants and researchers, so residual selection and performance bias cannot be excluded; these limitations, together with the small number of studies and wide confidence intervals, are reflected in the moderate‐to‐low GRADE certainty ratings across outcomes (Section 3.7) and should temper confidence in the pooled effect estimates. To reinforce the evidence base in this area, future investigations should prioritize the standardization of tDCS and assessment protocols so as to safeguard the validity of the conclusions. Finally, regarding safety, none of the included studies reported adverse events attributable to tDCS. However, it is unclear whether adverse events were systematically assessed using standardized instruments or merely not reported. This distinction is critical for interpreting the safety profile of tDCS in children with ASD. The absence of reported adverse events should not be interpreted as definitive evidence of safety; rather, future studies should prospectively and systematically assess tolerability and side effects using validated tools (e.g., structured adverse event checklists, discontinuation rates, participant‐reported discomfort) to establish a comprehensive safety profile for this pediatric population.

## 5 Conclusion

In summary, this meta‐analysis of seven RCTs (n = 235) provides preliminary evidence that tDCS, most commonly delivered at 1 mA over the DLPFC, is associated with improvements in some ASD‐related behavioral outcomes (ATEC, ABC) and a potential, low‐certainty benefit for sleep disturbances (CSHQ), whereas no significant effect was found on CARS. Given the moderate‐to‐low certainty of evidence across outcomes and the methodological limitations identified above, these findings should be regarded as preliminary rather than as establishing tDCS—or a specific 1‐mA DLPFC protocol—as an effective treatment for core ASD symptoms or sleep disturbances. Anodal tDCS stimulation of the left DLPFC may be a promising direction for future protocols, but larger, adequately powered, randomized double‐blind sham‐controlled trials using RoB 2 and gold‐standard, core‐symptom‐specific assessment methods are needed before firmer conclusions can be drawn.

## Author Contributions

M.W. and M.Y. participated in data analysis and drafted the original manuscript. M.W. was responsible for data collection and the literature review. Both M.W. and M.Y. contributed to manuscript review and editing. M.Y. oversaw project supervision and administration. All authors read and approved the final version of the manuscript.

## Funding

The authors have nothing to report.

## Ethics Statement

This article does not involve any studies conducted by the authors on human participants or animals.

## Conflicts of Interest

The authors declare no conflicts of interest.

## Data Availability

The raw data for this study can be obtained by contacting the corresponding author at [18590704645@163.com] or are publicly available in the appendix/online databases (if available) (https://www.cnki.net/ https://www.sinomed.ac.cn/zh/index.jsp?type=wx https://www.wanfangdata.com.cn/ https://qikan.cqvip.com/). We are committed to following relevant ethical regulations to ensure legal compliance of data sharing.
